# Comparison of urine albumin creatinine ratio with the pediatric index of mortality 2 score for prediction of pediatric intensive care unit outcomes

**DOI:** 10.1007/s11845-021-02755-4

**Published:** 2021-09-09

**Authors:** Shifa Nismath, Suchetha S. Rao, B. S. Baliga, Vaman Kulkarni, Gayatri M. Rao

**Affiliations:** 1 Department of Pediatrics, Kasturba Medical College, Mangalore, Manipal Academy of Higher Education, Manipal, India; 2 Department of Community Medicine, Kasturba Medical College, Mangalore, Manipal Academy of Higher Education, Manipal, India; 3 Department of Biochemistry, Kasturba Medical College, Mangalore, Manipal Academy of Higher Education, Manipal, India

**Keywords:** Critical illness, Microalbuminuria, Mortality, Multiple organ dysfunction syndrome, Prognosis

## Abstract

**Background:**

Predicting morbidity and mortality in a pediatric intensive care unit (PICU) is of extreme importance to make precise decisions for better outcomes.

**Aim:**

We compared the urine albumin creatinine ratio (ACR) with the established PICU score, pediatric index of mortality 2 (PIM 2) for predicting PICU outcomes.

**Methods:**

This cross-sectional study enrolled 67 patients admitted to PICU with systemic inflammatory response syndrome. Urine ACR was estimated on admission, and PIM 2 score was calculated. ACR was compared with PIM 2 for PICU outcome measures: the need for inotropes, development of multiple organ dysfunction syndrome (MODS), duration of PICU stay, and survival.

**Results:**

Microalbuminuria was found in 77.6% of patients with a median ACR of 80 mg/g. ACR showed a significant association with the need for inotropes (*p* < 0.001), MODS (*p* = 0.001), and significant correlation to PICU stay (*p* 0.001, rho = 0.361). The area under the receiver operating characteristic curve for ACR (0.798) was comparable to that of PIM 2 (0.896). The cutoff value of ACR derived to predict mortality was 110 mg/g. The study subjects were divided into 2 groups: below cutoff and above the cutoff. Outcome variables, inotrope use, MODS, mortality, and PICU stay compared between these subgroups, were statistically significant.

**Conclusion:**

ACR is a good predictor of PICU outcomes and is comparable to PIM 2 for mortality prediction.

## Introduction

Predicting morbidity and mortality in pediatric intensive care units is of extreme importance and can be challenging. Appropriate prediction helps to make the right decisions and thereby improve outcomes [1]. Many clinical scores like pediatric index of mortality 2 (PIM 2), pediatric risk of mortality (PRISM) score, and pediatric logistic organ dysfunction (PELOD) score are used to predict PICU outcomes [1, 2]. These scores have been validated in ICU settings of developed countries [3, 4]. Calculation of these scores requires many variables and internet facilities.

In critically ill patients, inflammatory response leads to endothelial membrane damage, a subsequent increase in capillary permeability results in transient albuminuria. The extent of albuminuria is variable, and most instances are not measured by urine dipstick protein estimation, hence known as microalbuminuria. The microalbuminuria can be measured by spot urine albumin creatinine ratio (ACR) [5]. The ACR is a simple, easy, and non-invasive measure that can be a good predictor of PICU outcome [6, 7].

We aimed to compare the ACR with the established PICU scoring system, PIM 2 as a predictor of PICU outcome. We decided to opt for PIM 2 as it is taken at 1 h from admission, which eliminates treatment-related changes in the scoring [4].

## Method

A cross-sectional, analytical study was conducted from January 2016 to September 2016, in tertiary care teaching hospitals affiliated to Kasturba Medical College Mangalore, Manipal Academy of Higher Education. Institutional ethics committee permission was obtained before the start of the study. Permission was obtained from the Medical Superintendent of the hospitals.

A sample size of 67 was calculated with a 95% confidence interval, 10% margin of error, and using data from a previous study by Basu et al. which showed a prevalence of 78% of microalbuminuria in the first 6 h of admission [8].

Patients with systemic inflammatory response syndrome admitted to PICU were included [9]. Patients with chronic renal disease, acute kidney injury, urinary tract infections, nephrotic syndrome, acute glomerulonephritis, and nephrotoxic drugs were excluded. A patient information sheet was given; the purpose of the study was explained, and written informed consent was obtained from parents.

Blood investigations including those required to calculate the PIM 2 score were sent on admission, and urine sample was collected within 1 h of admission by direct catch or by catheterization for estimation of albumin creatinine ratio. Variables used for PIM 2 score calculation were elective admission to the PICU, recovery post-procedure, admitted following cardiac bypass, high-risk diagnosis, low-risk diagnosis, no response of pupils to bright light, mechanical ventilation if required anytime during the first hour in PICU, systolic blood pressure mm Hg, base excess, and partial pressure of arterial oxygen (PaO_2_). PIM 2 score was calculated using an online calculator, and a predicted death rate was found [10].

Urine creatinine was estimated using the Jaffe method, and urinary albumin was estimated using a kit based on the immunoturbid metric method, using semi auto analyzer. Urine ACR was calculated. Urine ACR ratio 30–300 mg/g creatinine suggested microalbuminuria [5]. Patients were followed up subsequently until their PICU stay about the need for inotropes, development of multiple organ dysfunction syndrome (MODS), recovery, or mortality. The total duration of PICU stay was noted.

## Statistical analysis

The collected data was coded and entered on the Statistical Package for IBM (SPSS) Statistics for Windows version 25.0, Armonk, NY, IBM Corp. Descriptive data were expressed as percentages, mean, standard deviation, or median. Mann–Whitney *U* test was used to compare parameters between survivors and non-survivors. A *p* < 0.05 was considered statistically significant. The receiver operating characteristic (ROC) curve was constructed, and the area under the curve was calculated.

## Results

A total of 67 patients admitted to PICU were enrolled in the study. The median age of the participants was 2 years (IQR 0.41–6). The study comprised 52.2% males and 47.8% females. Most of the cases belonged to respiratory illness 31 (46.3%) like pneumonia and acute respiratory distress syndrome. Microalbuminuria was found in 52 patients (77.6%); median ACR was 80 (IQR 36. 31–236.14). Median PIM 2 score was 1.8 (IQR 1.2–2.2) Median PICU stay was 4 days (IQR 3–6). Six (8.9%) deaths were observed in PICU. A comparison of parameters between survivors and non-survivors is depicted in Table 1.

**Table 1:** Comparison of parameters between survivors and non-survivors

Variables	Non-survivors, *n* = 6 Median (IQR)	Survivors, *n* = 61 Median (IQR)	*p*
Hemoglobin (gm/dl)	8.60 (7.22–11.05)	10.00 (9.30–11.70)	0.109
Total counts (cells/cumm)	25,150 (12,000–34,375)	12,700 (6550–17,000)	0.025
Absolute neutrophil count (cells/cumm)	11,936 (10,960–15,864)	5600 (2514–11,377.5)	0.022
Platelet (cells/cumm)	224,500 (162,000–393,250)	334,000 (180,000–486,000)	0.345
Creatinine (mg/dl)	0.45 (0.30–1.02)	0.30 (0.30–0.50)	0.121
SGOT (U/l)	67.50 (39.00–99.00)	33 (22–47)	0.055
Prothrombin time (s)	19.60 (15.85–33.97)	15.50 (14.80–16.00)	0.016
INR	1.47 (1.20–2.96)	1.20 (1.09–1.40)	0.161
Total bilirubin (mg/dl)	0.59 (0.33–1.01)	0.32 (0.21–0.56)	0.227
Calcium (mg/dl)	9.05 (7.82–9.40)	9.00 (8.80–9.45)	0.815
Potassium (mmol/l)	4.36 (3.94–4.84)	4.60 (4.20–5.07)	0.339
Bicarbonate (mmol/l)	11.85 (6.80–15.65)	16.80 (14.35–19.20)	0.017
PaO_2_ (mmHg)	86.90 (51.57–137.50)	98 (90–110.5)	0.402
Base excess (mmol/l)	−10.90 (−26.10– − 2.75)	−4.00 (−5.50– −2.00)	0.135
ACR (mg/g)	361.50 (191.00–664.25)	70.36 (32.80–196.00)	0.017
PIM2	23.20 (2.55–55.85)	1.70 (1.20–2.10)	0.01
PICU days	6 (3–17.25)	2 (2–5)	0.012

*IQR* interquartile range, *SGOT* serum glutamic oxaloacetic transaminase, *PaO*_*2*_ partial pressure of oxygen, *INR* international normalized ratio, *ACR* albumin creatinine ratio, *PIM2* pediatric index of mortality 2, *PICU* pediatric intensive care unit

**Fig. 1:**
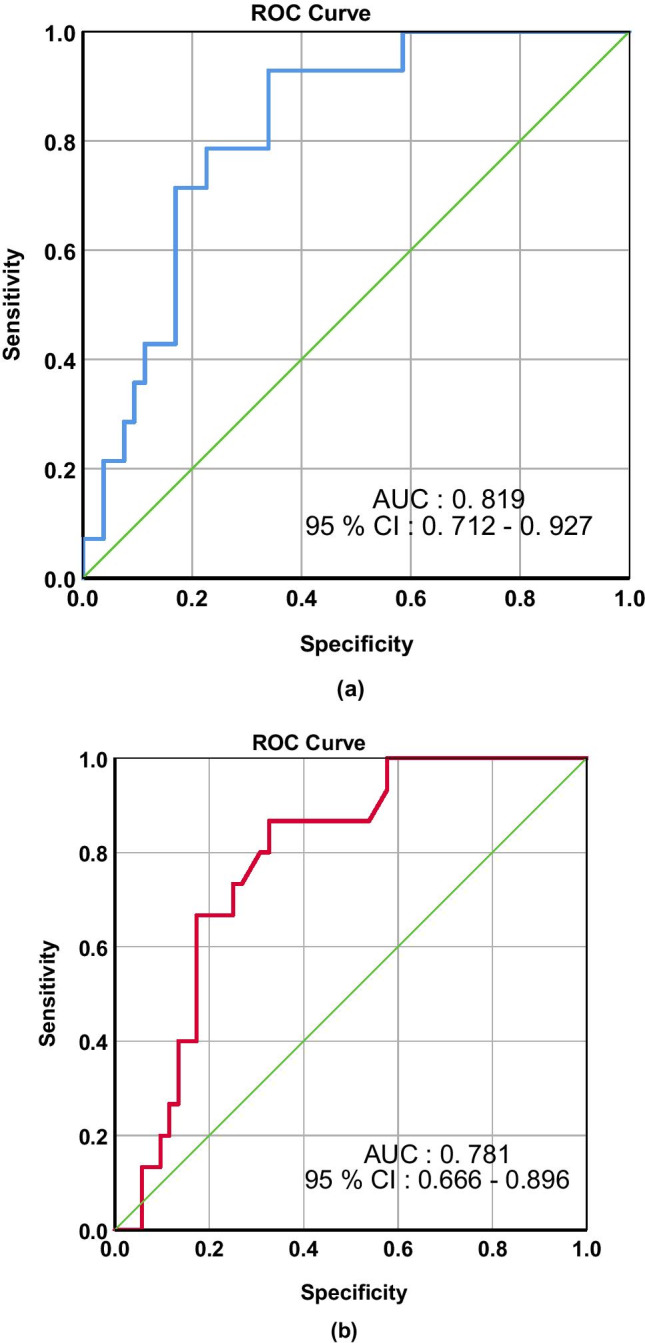
Receiver operating characteristic curve of albumin creatinine ratio at admission for the need of inotrope (**a**) and multiple organ dysfunction syndrome (**b**) in the pediatric intensive care unit

ACR had a significant correlation to PICU stay (*p* 0.001, rho = 0.361). Among the study subjects, 14 (20.9%) required inotropes and 15 (22.4%) developed MODS. ACR showed a statistically significant association with the need for inotropes (*p* < 0.001) and the development of MODS (*p* = 0.001). ROC curve for ACR and need of inotrope and MODS are shown in Fig. 1a, b, respectively. AUC for the need of inotrope was 0.819, and MODS was 0.781, respectively (Fig. 1a, b).

**Fig. 2:**
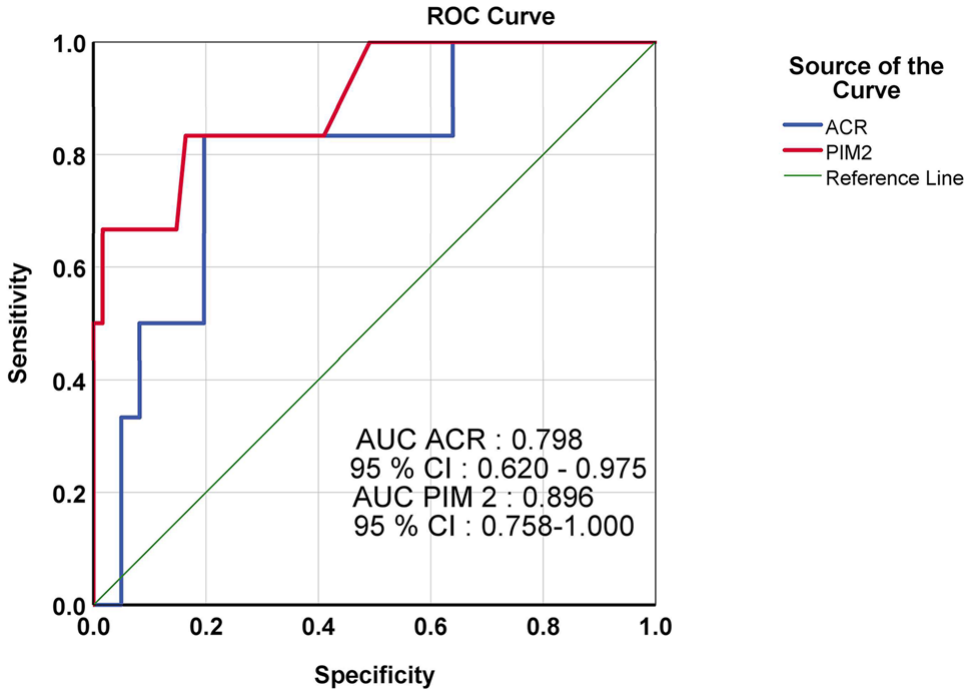
Receiver operating characteristic curves of albumin creatinine ratio at admission and pediatric index of mortality 2 score for mortality in pediatric intensive care unit

As per the ROC curve, AUC derived for mortality for ACR was 0.798, and AUC for mortality for PIM 2 was 0.896. The AUC for ACR was comparable to the standard clinical mortality score PIM 2 (Fig. 2). The cutoff value of ACR derived to predict mortality was 110 mg/g and had a sensitivity of and specificity of 83.3% and 41.1%. Based on this cutoff value, the study subjects were divided into 2 groups: below cutoff (≤ 110 mg/g) and above cutoff (> 110 mg/g). Outcome variables such as the need for inotropes, development of MODS, mortality, and median PICU stay days compared between these subgroups were statistically significant (Table 2).

**Table 2:** Comparison of the pediatric intensive care unit outcome parameters with albumin creatinine ratio 110 mg/g cutoff

Parameter	ACR ≤ 110 mg/g	ACR > 110 mg/g	*p* value
	*n* = 39 *n* (%)	*n* = 28 *n* (%)	
Inotrope use Present Absent	3 (7.7) 36 (92.3)	11 (39.3) 17 (60.7)	0.002
Multiple organ dysfunction syndrome Present Absent	3 (7.7) 36 (92.3)	12 (42.9) 16 (57.1)	0.001
Outcome Expired Survived	1 (2.6) 38 (97.4)	5 (17.9) 23 (82.1)	0.042
PICU stay days Median (IQR)	3 (3–5)	5.5 (3.5–7)	0.004

*ACR* albumin creatinine ratio, *PICU* pediatric intensive care unit, *IQR* interquartile range

## Discussion

Systemic inflammation is associated with increased permeability to plasma proteins which usually returns to normal within 48 h with the improvement in the disease process. As renal tubules have limited capacity to handle albumin reabsorption, increased capillary permeability leads to increased urinary albumin excretion [11].

In the present study, ACR was calculated within 1 h of admission, which showed the presence of MA in 77.6% of patients. A study by Ayse et al. reported the prevalence of MA in 64% of critically ill children. They found that MA was much higher in children with sepsis. They also concluded that ACR at admission as well as at 24 h had a good correlation with clinical scores (PELOD, PRISM, and PIM 2) for mortality prediction. ACR levels were associated with the duration of mechanical ventilation, the need for inotropic support, and the extent of MODS [5]. A study by Basu et al. found that 78% admitted to ICU had microalbuminuria with a median value of 125.6 mg/g; 24 h of admission microalbuminuria persisted in 67%; however, the median value had reduced to 62.6 mg/g [8]. Hence, the timing of sampling of urine to determine the ACR is crucial. With the onset of recovery, a rapid decline in ACR may be seen. Median ACR values were much higher in patients with sepsis and non-survivors [8].

Sachdev et al. studied the role of urinary ACR in children with varying severity of sepsis admitted to PICU. The study enrolled 138 participants, and serial ACR values were estimated. The ACR showed a rising trend with the increasing severity of sepsis. ACR values were greater in non-survivors. They concluded ACR > 102 mg/g correlated well with the lengthier duration of mechanical ventilation, necessity for inotropes, and death. ACR showed a good correlation with PELOD and PRISM scores [6].

In the present study, ACR showed a good correlation with PIM 2 score. The cutoff value of ACR derived to predict mortality was 110 mg/g which is comparable to previous studies [6, 12, 13]. In the present study, need for inotrope, presence of MODS, mortality, and duration of PICU stay were significant in ACR > 110 mg/g group.

A study by Thorevska in critically ill medical patients reported a prevalence of MA in 69% of the patients. ACR ≥ 100 mg/g predicted mortality and hospital stay independently. Risk of death increased by 2.7 times when ACR was > 100 mg/g [13].

A study by Gosling et al. which recruited medical and surgical patients in adult ICU concluded that ACR predicted ICU mortality and inotrope requirement better than APACHE II and SOFA score [14]. Tayeh et al. reported that ACR at 24 h of admission was higher in patients who were mechanically ventilated, those requiring inotropes. Increasing ACR was found to be a predictor of mortality [15].

### Limitations

ACR was done only on admission. Repeating ACR subsequently and comparing it with patient recovery or worsening give a better idea of the predictive ability of ACR. The sample size of the present study was small. Studies with larger sample size help to generalize the results. For the prediction of PICU mortality, PIM 2 score was used instead of the updated version of PIM 3.

## Conclusion

ACR was found to have significant relation to the need for inotropes, development of MODS, and prediction of mortality similar to PIM 2. In addition, ACR had a good correlation to the duration of the PICU stay. To conclude, ACR is an easy, cost-effective, and reliable test for predicting morbidity and mortality in a PICU setting.

## Abbreviations

ACR: Albumin creatinine ratio
AUC: Area under the curve
INR: International normalized ratio
IQR: Interquartile range
MODS: Multiple organ dysfunction syndrome
Pa02: Partial pressure of arterial oxygen
PELOD: Pediatric logistic organ dysfunction sore
PICU: Pediatric intensive care unit
PIM 2: Pediatric index of mortality 2
PRISM: Pediatric risk of mortality
ROC: Receiver operating characteristic
SGOT: Serum glutamic oxaloacetic transaminase

## References

[1] Gulla KM, Sachdev A (2016). Illness severity and organ dysfunction scoring in pediatric intensive care unit. Indian J Crit Care Med.

[2] Gandhi J, Sangareddi S, Varadarajan P (2013). Pediatric index of mortality 2 score as an outcome predictor in pediatric intensive care unit in India. Indian J Crit Care Med.

[3] Slater A, Shann F (2004). The suitability of the pediatric index of mortality (PIM), PIM2, the pediatric risk of mortality (PRISM), and PRISM III for monitoring the quality of pediatric intensive care in Australia and New Zealand. Pediatr Crit Care Med.

[4] Slater A, Shann F, Pearson G (2003). PIM2: a revised version of the paediatric index of mortality. Intensive Care Med.

[5] Anil AB, Anil M, Yildiz M et al (2014) The importance of microalbuminuria in predicting patient outcome in a PICU. Pediatr Crit Care Med 15:e220–5 10.1097/PCC.0000000000000113 24892488

[6] Sachdev A, Raheja K, Gupta N (2020). Association of urinary albumin: creatinine ratio with outcome of children with sepsis. Indian J Crit Care Med.

[7] Basu S, Chaudhuri S, Bhattacharyya M (2010). Microalbuminuria: an inexpensive, noninvasive bedside tool to predict outcome in critically ill patients. Indian J Clin Biochem.

[8] Basu S, Bhattacharya M, Chatterjee T (2010). Microalbuminuria: a novel biomarker of sepsis. Indian J Crit Care Med.

[9] Goldstein B, Giroir B, Randolph A (2005) International consensus conference on pediatric sepsis. International pediatric sepsis consensus conference: definitions for sepsis and organ dysfunction in pediatrics. Pediatr Crit Care Med 6:2–8 10.1097/01.PCC.0000149131.72248.E6 15636651

[10] Societe Francaise d’ Anesthesie et de Reanimation (2003) Scoring systems for ICU and surgical patients: PIM 2 (Paediatric Index of Mortality). https://sfar.org/scores2/pim22.php. Accessed 10 July 2021

[11] Fleck A, Raines G, Hawker F (1985). Increased vascular permeability: a major cause of hypoalbuminemia in disease and injury. Lancet.

[12] Nismath S, Rao SS, Baliga BS (2020). Comparative validity of microalbuminuria versus clinical mortality scores to predict pediatric intensive care unit outcomes. Clin Exp Pediatr.

[13] Thorevska N, Sabahi R, Upadya A (2003). Microalbuminuria in critically ill medical patients: prevalence, predictors, and prognostic significance. Crit Care Med.

[14] Gosling P, Czyz J, Nightingale P (2006). Microalbuminuria in the intensive care unit: clinical correlates and association with outcomes in 431 patients. Crit Care Med.

[15] Tayeh O, Taema KM, Eldesouky MI (2016). Urinary albumin/creatinine ratio as an early predictor of outcome in critically-ill septic patients. Egypt J Crit Care Med.

